# Changes in the Phenotypes of *Salmonella* spp. in Japanese Broiler Flocks

**DOI:** 10.14252/foodsafetyfscj.D-24-00001

**Published:** 2024-05-31

**Authors:** Yoshika Momose, Yoshimasa Sasaki, Kenzo Yonemitsu, Makoto Kuroda, Tetsuya Ikeda, Masashi Uema, Yoko Furuya, Hajime Toyofuku, Shizunobu Igimi, Tetsuo Asai

**Affiliations:** 1 Division of Biomedical Food Research, National Institute of Health Sciences, 3-25-26 Tonomachi, Kawasaki-ku, Kawasaki, Kanagawa 210-9501, Japan; 2 Division of Veterinary Science, Department of Veterinary Medicine, Obihiro University of Agriculture and Veterinary Medicine, Inada-cho, Obihiro, Hokkaido 080-8555, Japan; 3 Management Department of Biosafety, Laboratory Animal, and Pathogen Bank, National Institute of Infectious Diseases, 4-7-1, Gakuen, Musashimurayama, Tokyo 208-0011, Japan; 4 Pathogen Genomics Center, National Institute of Infectious Diseases, 1-23-1 Toyama, Shinjuku-ku,Tokyo 162-8640, Japan; 5 Department of Infectious Diseases, Hokkaido Institute of Public Health, Kita-19, Nishi-12, Kita-ku, Sapporo, Hokkaido 060-0819, Japan; 6 Japan Food Safety Verification Organization, 1-7-5, Sekiguchi, Bunkyo-ku, Tokyo 112-0014, Japan; 7 The United Graduate School of Veterinary Science, Yamaguchi University, 1677-1 Yoshida,Yamaguchi 753–8515, Japan; 8 Department of Applied Biology and Chemistry, Tokyo University of Agriculture, 1-1-1, Sakuragaoka,Setagaya-ku, Tokyo 156-8502, Japan; 9 The United Graduate School of Veterinary Science, Gifu University, 1-1, Yanagido, Gifu, Gifu 501-1193, Japan

**Keywords:** breast meat, broiler, cross-contamination, *Salmonella*, third-generation cephalosporin resistance

## Abstract

*Salmonella* infections represent a leading cause of foodborne illnesses; resistance to third-generation cephalosporins (TGCs), which are a first-choice antimicrobial for treating human *Salmonella* enteritis, has become a serious public health concern worldwide. Because the consumption of undercooked chicken meat products is a major cause of foodborne salmonellosis in Japan, we conducted three surveys at different periods between 2017 and 2022, with the cooperation of four abattoirs (two in Eastern and two in Western Japan). The first survey was conducted at abattoir A, which is located in Eastern Japan. *Salmonella* was detected in 84.4% of broiler flocks tested (27/32); among them, all the TGC-resistant isolates obtained from one farm (farm FA) were identified as *S.* Infantis. *Salmonella* was recovered from 62.5% of breast meat samples (20/32), with one case suggesting cross-contamination. The second survey was conducted at three other abattoirs to examine the prevalence of TGC-resistant *Salmonella*, in both Western (abattoirs B and C) and Eastern (abattoir D) Japan. *Salmonella* was detected in 90.6% of broiler flocks examined (29/32). TGC-resistant *S.* Infantis was isolated from 2 flocks until 2018 and not thereafter. Subsequently, isolates were identified as TGC-susceptible *S.* Schwarzengrund in both regions. The third survey was performed at abattoir A to elucidate whether there were changes in the phenotypes. Of the 11 broiler flocks introduced from farm FA, 10 were positive for *Salmonella* (90.9%); all the isolates were *S.* Schwarzengrund susceptible to TGC. This study shows that TGC-susceptible *S.* Schwarzengrund has replaced the resistant phenotypes among broiler flocks in both Eastern and Western Japan. Although chicken meat products could be cross-contaminated with *Salmonella* during the slaughtering process, reducing the prevalence of *Salmonella* in broiler flocks remains important to decrease *Salmonella* enteritis in humans.

## 1. Introduction

*Salmonella* is an enteric bacterial pathogen that causes foodborne illnesses in humans worldwide. In Japan, Kumagai *et al.* estimated the national burden of gastroenteritis caused by *Salmonella* species in 2011^1^^)^ and found that the annual incidence was approximately 31.7 cases per 100,000 population. The calculated disability-adjusted life years (DALYs) lost, based on the incidence estimates along with disability weights, were 3,145 DALYs. Foodborne diseases caused by *Campylobacter* species and enterohemorrhagic *Escherichia coli* have led to estimated losses of 6,099 and 463 DALYs, respectively. Similarly, *Salmonella* infections may represent a serious problem leading to social and economic losses.

Although antimicrobial therapy is generally not recommended for acute self-limiting enteritis, the administration of fluoroquinolones and third-generation cephalosporins (TGCs) may be effective for treating patients with severe symptoms and in health risk groups^2^^,^^3^^)^. In Japan, TGC administration is not approved in the poultry industry; however, off-label use was performed at a few hatcheries until it was voluntarily ceased in 2012^4^^,^^5^^)^. A nationwide survey conducted between 2007 and 2010 showed that 26.3% of *Salmonella* isolates from broiler flocks were resistant to the TGC, ceftiofur^6^^)^. Duc *et al.* found that the prevalence of TGC resistance in *Salmonella* in broiler chickens decreased from 52.7% to 25.8% in Kagoshima Prefecture (Southern Japan) after the withdrawal of TGC administration^7^^,^^8^^)^. Other studies reported that the prevalence of TGC resistance in *Salmonella* had increased in domestic retail chicken meat products until 2012^9^^,^^10^^)^ and decreased thereafter^10^^)^. This is consistent with findings from Canada, where there was a decrease in the prevalence of ceftiofur resistance in *E. coli* and *S.* Heidelberg isolated from retail chicken meat products after the withdrawal of ceftiofur administration^11^^)^.

From the perspective of food safety, it is important to continue monitoring the prevalence and antimicrobial resistance profiles of *Salmonella* in foods, especially chickens and their products, because most cases of human salmonellosis are foodborne^1^^)^. They are associated in part with the consumption of raw/undercooked contaminated poultry products^12^^,^^13^^)^. In this study, we conducted three surveys at different periods between 2017 and 2022, with the cooperation of four abattoirs, to determine whether the decreased prevalence of TGC resistance in *Salmonella* is observed in both Eastern and Western Japan.

## 2. Materials and Methods

### 2.1 Sample Collection

Three surveys were conducted in this study (**Table 1**). The first survey was conducted between December 2017 and February 2018. Cecal contents and breast meat samples were collected from broiler chickens on 16 different days at abattoir A, which is located in Eastern Japan. On each day of sample collection, the cecal contents were obtained from three broilers each of the first two consecutive flocks. One package of breast meat sample (weighing 2 kg) was also obtained from each flock, which comprised six pieces of half breast meat. The second survey was conducted at three other abattoirs (B and C in Western, and D in Eastern Japan) between November 2018 and November 2020. Cecal contents were collected from three broiler chickens each of 32 flocks, which were transported from different farms to abattoir B, C, or D. The third survey was conducted between October 2021 and October 2022 at abattoir A; cecal contents were collected from three broiler chickens each of 11 flocks, which were transported from farm FA. Farm FA was the largest farm surveyed in this study, containing 29 broiler houses. It shipped one flock approximately once every 2 days to abattoir A. Flocks from farm FA account for 27% of the poultry slaughtered annually at abattoir A.

**Table 1.** Summary of the three surveys in this study.

	Year	Abattoir		Samples		No. included	
		Code	Location	Type	No. tested	farms	flocks
1st survey	2017-2018	A	Eastern Japan	Cecal contents	96	15	32 (27)^a^
				Breast meat	32	15	32 (20)
2nd survey	2018-2020	B	Western Japan	Cecal contents	51	17	17 (17)
		C	Western Japan	Cecal contents	24	8	8 (7)
		D	Eastern Japan	Cecal contents	21	7	7 (5)
3rd survey	2021-2022	A	Eastern Japan	Cecal contents	33	1	11 (10)

^a^ The values in parentheses refer to the number of flocks positive for *Salmonella* spp.

### 2.2 Isolation of *Salmonella* spp.

Isolation of *Salmonella* spp. was carried out according to the Japanese standard method, NIHSJ-01^14^^,^^15^^)^, with some modifications. Approximately 1 g of each cecal content and 150 g of each breast meat sample were placed in a buffered peptone water (Eiken Chemical Co., Ltd., Tokyo, Japan) of nine-fold volume to prepare a 1:10 suspension. The cecal and breast meat suspension was homogenized using VORTEX-GENIE 2 (Scientific Industries, Inc., Bohemia, NY, USA) and a Masticator (IUL, S.A., Barcelona, Spain), respectively, followed by incubation at 37 °C for 22 h ± 2 h. Aliquots of 0.1 and 1 mL of each culture were transferred to 10 mL of Rappaport-Vassiliadis broth (Oxoid Ltd., Basingstoke, Hampshire, UK) and 9 mL of tetrathionate broth base, Hajna (Eiken Chemical), respectively, and incubated at 42 °C for 22 h ± 2 h. Xylose-lysine-deoxycholate agar (Kanto Chemical Co., Inc., Tokyo, Japan) and CHROMagar Salmonella (CHROMagar, Paris, France) were used for selective isolation. After incubation at 37 °C for 22 h ± 2 h, typical or suspected colonies were purified and identified biochemically^14^^,^^15^^)^. Serotyping was performed according to the Kauffmann-White scheme^16^^)^ using somatic and flagellar antisera (Denka Co., Ltd., Tokyo, Japan). A flock was considered positive for *Salmonella* when *Salmonella* was isolated from at least one sample of cecal contents. One isolate from each serovar was selected per flock and subjected to subsequent antimicrobial susceptibility testing.

### 2.3 Antimicrobial Susceptibility Testing

Antimicrobial susceptibility testing was performed using the broth microdilution method with 96-well microtiter plates (Eiken Chemical). The following 12 antimicrobial agents were examined: ampicillin, cefazolin, cefotaxime, streptomycin, gentamicin, kanamycin, tetracycline, nalidixic acid, ciprofloxacin, colistin, chloramphenicol, and trimethoprim. The resistance breakpoints were determined in accordance with the guidelines by Clinical and Laboratory Standards Institute^17^^)^ and National Veterinary Assay Laboratory of Japan^18^^)^. *E. coli* ATCC 25922 was used as the quality control strain.

### 2.4 Genetic Analysis of *Salmonella* Isolates

Cefotaxime-resistant isolates were analyzed to detect plasmid-mediated AmpC β-lactamase genes using multiplex polymerase chain reaction^19^^)^, and sequence analysis was subsequently performed with primer pairs reported by Noda *et al.*^20^^)^ on a 3730xl DNA Analyzer (Thermo Fisher Scientific, Waltham, MA, USA). When cross-contamination was suspected in the first survey, the relevant isolates were subjected to whole-genome sequencing (WGS) analysis^21^^)^ to determine the antimicrobial resistance genes and sequence types based on multi-locus sequence typing (MLST). Sequencing libraries for each isolate were prepared using the QIAseq FX Library Kit (QIAGEN N.V., Venlo, The Netherlands) to obtain paired-end sequences (300 bp × 2) on the Illumina MiSeq platform (Illumina, Inc., San Diego, CA, USA). The draft genome sequence was assembled using A5-miseq^22^^)^ and gene annotation was performed using DFAST version 1.2.3, with the following databases: DFAST default database^23^^)^, ResFinder database^24^^)^, and Bacterial Antimicrobial Resistance Reference Gene database (PRJNA313047). MLST was performed in accordance with the protocols available in the MLST database (https://pubmlst.org/).

## 3. Results and Discussion

At the time of the start of this study in 2017, five years had already passed since TGC administration was ceased voluntarily in the poultry industry in Japan. The first survey was conducted at abattoir A in Eastern Japan; 27 out of 32 broiler flocks were positive for *Salmonella* (84.4%), and 29 isolates were eventually obtained (**Table 2**). The most frequent serovar was *S.* Infantis, followed by *S.* Schwarzengrund. Nine isolates of *S*. Infantis were resistant to cefotaxime, a TGC, and harbored *bla*_CMY-2_ as the resistance gene. They were isolated from 9 flocks introduced from one farm (farm FA). Farm FA did not use the “all-in/all-out” system at the farm level, whereas the other 14 farms included in the first survey used this system. These findings suggest that chicks colonized by TGC-resistant *Salmonella* were not introduced into the broiler farms at around the time of the study; however, they were introduced before and survived while moving from one broiler house to another in farm FA. The second survey was conducted to examine the prevalence of TGC-resistant *Salmonella*, in both Western (abattoirs B and C) and Eastern (abattoir D) Japan, because a regional difference in the serological distribution of *Salmonella* was observed in broiler chickens in a previous study^6^^)^. In total, 17 out of 17 flocks at abattoir B (100%), 7 out of 8 flocks at abattoir C (87.5%), and 5 out of 7 flocks at abattoir D (71.4%) were positive for *Salmonella*, and 29 isolates were eventually obtained (**Table 2**). The most frequent serovar was *S.* Schwarzengrund, which was susceptible to TGC in both regions. Two strains of cefotaxime-resistant *S*. Infantis harboring *bla*_CMY-2_ were isolated from the cecal contents obtained from different broiler farms in 2018 slaughtered in abattoir B. No cefotaxime-resistant *Salmonella* was isolated in 2019 or later, and cefotaxime-susceptible *S.* Schwarzengrund was predominantly isolated from three abattoirs, including abattoir B. This may be because the two farms reused litter, although they use the “all-in/all-out” system at a farm level, and pre-existing TGC-resistant *Salmonella* survived in the surroundings for a while longer than other farms. According to the results of the second survey, TGC-susceptible *S*. Schwarzengrund was considered to have recently replaced TGC-resistant *S*. Infantis in Japanese broiler flocks. Based on this hypothesis, we investigated the presence of TGC-resistant *S*. Infantis once again in broiler flocks introduced from farm FA at abattoir A in the third survey. Of the 11 broiler flocks tested from 2021 to 2022, 10 were positive for *Salmonella* (90.9%), and 10 isolates were eventually obtained; cefotaxime-susceptible and kanamycin-resistant *S.* Schwarzengrund was predominant (**Table 2**). Cefotaxime-resistant *Salmonella* was never isolated during the third survey. The prevalence of TGC resistance in *Salmonella* isolates was 31.0% in the first, 6.9% in the second, and 0% in the third survey.

**Table 2.** Serovars and antimicrobial resistance profiles of *Salmonella* isolated from the cecal contents of broilers in the first, second, and third surveys.

Serovar	Antimicrobial resistance profile ^a^	1st survey in 2017–2018 (n = 29)		2nd survey in 2018–2020 (n = 29)			3rd survey in 2021–2022 (n = 10)	
		Abattoir	(Farm FA)	Abattoir			Abattoir	(Farm FA)
		A		B	C	D	A	
*S*. Infantis	ABPC + CEZ + CTX + SM + TC + TMP	9 ^b^	(9) ^c^	‒ ^d^	‒	‒	‒	(-)
	ABPC + CEZ + CTX + SM + TC	‒	(-)	1 *	‒	‒	‒	(-)
	ABPC + CEZ + CTX + TC	‒	(-)	1 *	‒	‒	‒	(-)
	ABPC + KM + TMP	‒	(-)	1	‒	‒	‒	(-)
	SM + KM + TC + NA + TMP	1	(-)	‒	‒	‒	‒	(-)
	SM + KM + TC	2	(-)	‒	‒	‒	‒	(-)
	TC + TMP	1	(-)	‒	‒	‒	‒	(-)
*S*. Schwarzengrund	SM + KM + TC + NA + TMP	‒	(-)	‒	1	‒	‒	(-)
	SM + KM + TC + TMP	2	(-)	3	4	3	‒	(-)
	SM + KM + TC	‒	(-)	1	1	1	‒	(-)
	SM + TC + TMP	‒	(-)	5	‒	1	‒	(-)
	SM + TC	‒	(-)	4	‒	‒	‒	(-)
	SM + KM	‒	(-)	‒	‒	‒	1	(1)
	KM + NA	‒	(-)	‒	‒	‒	2	(2)
	KM + TMP	‒	(-)	‒	1	‒	‒	(-)
	KM	7	(-)	‒	‒	‒	7	(7)
*S*. Anatum	Susceptible	3	(2)	‒	‒	‒	‒	(-)
*S*. Bareilly	Susceptible	2	(2)	‒	‒	‒	‒	(-)
*S*. Typhimurium	ABPC + SM + KM + TC + NA + TMP	1	(-)	‒	‒	‒	‒	(-)
Untypable	SM + KM + TC + NA + TMP	1	(-)	‒	‒	‒	‒	(-)
	SM + TC + TMP	‒	(-)	1	‒	‒	‒	(-)
Total		29	(13)	17	7	5	10	(10)

^a^ ABPC; ampicillin, CEZ; cefazolin, CTX; cefotaxime, SM; streptomycin, KM; kanamycin, TC; tetracycline, NA; nalidixic acid, TMP; trimethoprim.

^b^ The values refer to the number of isolates.

^c^ The values in parentheses refer to the number of isolates from farm FA out of the total.

^d^ ‒; not detected.

*; isolate in 2018.

To elucidate whether cross-contamination occurs during the slaughtering process, *Salmonella* was also isolated from the breast meat samples collected in the first survey. Twenty-three isolates were obtained from breast meat samples derived from 19 *Salmonella*-positive and one *Salmonella*-negative flocks (Table 3); 18 isolates exhibited the same serovars and antimicrobial resistance profiles as those from the cecal contents of the corresponding flocks. Flocks B21 and B22 were introduced from different farms and slaughtered consecutively on the same day; *S.* Infantis exhibiting the same antimicrobial resistance profile was isolated from the breast meats from both flocks. The same phenotype was also obtained from the cecal contents of flock B21, whereas *Salmonella* was not isolated from those of flock B22. To gain more information on these three isolates, they were subjected to WGS analysis. These isolates harbored the same antimicrobial resistance genes and were classified into the same sequence type (Table 4). Although the MLST technique does not thoroughly distinguish genomic heterogeneity among *S.* Infantis strains^30^^)^, the three strains could not have been differentiated from each other based on the serological and antimicrobial characteristics. We previously reported that chicken meat products derived from a *Campylobacter*-negative flock could be cross-contaminated by a *Campylobacter-*positive flock slaughtered right before^25^^,^^26^^)^. As both *Campylobacter* and *Salmonella* colonize the intestinal tract of broilers, and several broiler flocks from different farms are slaughtered consecutively in a single abattoir, cross-contamination of chicken meat products with *Salmonella* could occur in a similar manner to *Campylobacter* contamination. Nevertheless, because of the limited number of breast meat samples included in this study, intensive investigation is needed to determine how frequently cross-contamination occurs during the slaughtering process.

**Table 3.** Comparison of *Salmonella* isolates obtained from cecal contents and breast meat of broilers in the first survey.

	Flock	Farm	Cecal contents (n = 29)		Breast meat (n = 23)	
			Serovar	Antimicrobial resistance profile ^a^	Serovar	Antimicrobial resistance profile
Day 1	B1	FA	*S.* Infantis	ABPC + CEZ + CTX + SM + TC + TMP	*S.* Infantis	ABPC + CEZ + CTX + SM + TC + TMP
	B2	FB	*S.* Schwarzengrund	KM	*S.* Schwarzengrund	KM
Day 2	B3	FA	*S.* Infantis	ABPC + CEZ + CTX + SM + TC + TMP	*S*. Infantis	ABPC + CEZ + CTX + SM + TC + TMP
			*S.* Anatum	susceptible	*S.* Schwarzengrund	SM + KM + TMP
	B4	FC	*S.* Anatum	susceptible	*S.* Infantis	SM + KM + TC + NA + TMP
Day 3	B5	FA	‒ ^b^	‒	‒	‒
	B6	FD	*S.* Schwarzengrund	KM	*S.* Schwarzengrund	KM
Day 4	B7	FE	*S.* Schwarzengrund	KM	*S.* Schwarzengrund	KM
	B8	FA	*S.* Bareilly	susceptible	‒	‒
Day 5	B9	FA	*S.* Infantis	ABPC + CEZ + CTX + SM + TC + TMP	*S.* Infantis	ABPC + CEZ + CTX + SM + TC + TMP
	B10	FF	*S.* Infantis	TC + TMP	‒	‒
Day 6	B11	FA	*S.* Anatum	susceptible	*S.* Anatum	susceptible
	B12	FG	*S.* Infantis	SM + KM + TC	‒	‒
Day 7	B13	FA	*S.* Infantis	ABPC + CEZ + CTX + SM + TC + TMP	‒	‒
	B14	FH	*S.* Schwarzengrund	KM	*S.* Schwarzengrund	KM
Day 8	B15	FA	*S.* Infantis	ABPC + CEZ + CTX + SM + TC + TMP	‒	‒
	B16	FG	*S.* Infantis	SM + KM + TC	‒	‒
Day 9	B17	FA	*S.* Infantis	ABPC + CEZ + CTX + SM + TC + TMP	*S.* Infantis	ABPC + CEZ + CTX + SM + TC + TMP
	B18	FI	‒	‒	‒	‒
Day 10	B19	FA	*S.* Infantis	ABPC + CEZ + CTX + SM + TC + TMP	*S.* Infantis	ABPC + CEZ + CTX + SM + TC + TMP
					*S.* Anatum	susceptible
	B20	FI	*S.* Schwarzengrund	KM	*S.* Schwarzengrund	KM
Day 11	B21	FA	*S.* Infantis	ABPC + CEZ + CTX + SM + TC + TMP	*S.* Infantis	ABPC + CEZ + CTX + SM + TC + TMP
	B22	FJ	‒	‒	*S.* Infantis	ABPC + CEZ + CTX + SM + TC + TMP
Day 12	B23	FA	‒	‒	‒	‒
	B24	FK	*S.* Schwarzengrund	KM	*S.* Schwarzengrund	KM
Day 13	B25	FA	*S.* Infantis	ABPC + CEZ + CTX + SM + TC + TMP	*S.* Infantis	ABPC + CEZ + CTX + SM + TC + TMP
					*S.* Anatum	susceptible
	B26	FC	*S.* Schwarzengrund	KM	*S.* Schwarzengrund	KM
Day 14	B27	FL	*S.* Infantis	SM + KM + TC + NA + TMP	*S.* Infantis	SM + KM + TC + NA + TMP
			Untypable	SM + KM + TC + NA + TMP		
	B28	FM	‒	‒	‒	‒
Day 15	B29	FN	*S.* Typhimurium	ABPC + SM + KM + TC + NA + TMP	‒	‒
	B30	FO	*S*. Schwarzengrund	SM + KM + TC + TMP	*S*. Schwarzengrund	SM + KM + TC + TMP
Day 16	B31	FA	*S.* Bareilly	susceptible	‒	‒
	B32	FN	*S.* Schwarzengrund	SM + KM + TC + TMP	*S.* Schwarzengrund	SM + KM + TC + TMP

^a^ ABPC; ampicillin, CEZ; cefazolin, CTX; cefotaxime, SM; streptomycin, KM; kanamycin, TC; tetracycline, NA; nalidixic acid, TMP; trimethoprim.

^b^ ‒; not detected.

**Table 4.** Genetic characteristics of three strains of *S.* Infantis isolated from flocks B21 and B22.

Flock	Farm	Sample	Sequence type	Antimicrobial resistance profile ^a^	Antimicrobial resistance genes
B21	FA	Cecal contents	32	ABPC + CEZ + CTX + SM + TC + TMP	*aac(6′)-Iaa*, *ant(3′′)-Ia*, *bla*_CMY-2_, *dfrA14*, *qacEdelta1*, *sul1*, *tet(A)*
		Breast meat	32	ABPC + CEZ + CTX + SM + TC + TMP	*aac(6′)-Iaa*, *ant(3′′)-Ia*, *bla*_CMY-2_, *dfrA14*, *qacEdelta1*, *sul1*, *tet(A)*
B22	FJ	Cecal contents	‒	‒	‒
		Breast meat	32	ABPC + CEZ + CTX + SM + TC + TMP	*aac(6′)-Iaa*, *ant(3′′)-Ia*, *bla*_CMY-2_, *dfrA14*, *qacEdelta1*, *sul1*, *tet(A)*

Nucleotide sequence data reported are available in the DDBJ Sequence Read Archive under the accession numbers DRR524581 (the isolate from the cecal contents, flock B21), DRR524582 (the isolate from the breast meat, flock B21), and DRR524583 (the isolate from the breast meat, flock B22).

^a^ ABPC; ampicillin, CEZ; cefazolin, CTX; cefotaxime, SM; streptomycin, TC; tetracycline, TMP; trimethoprim.

^b^ ‒; not detected.

In the first decade of the 2000s, *S.* Infantis was the most frequent isolate in broiler chickens^27^^,^^28^^,^^29^^)^, and the resistance rate to ceftiofur was low (1.8%)^29^^)^. The ceftiofur-resistance rate of *Salmonella* isolated from broiler flocks was 26.3%, especially that of *S.* Infantis was 38.1%, according to a nationwide survey conducted between 2007 and 2010^6^^)^. Duc *et al.* showed that the increased prevalence of TGC-susceptible *S*. Schwarzengrund led to a decrease in the prevalence of TGC-resistant *Salmonella* in Southern Japan^7^^,^^8^^)^. This study has some limitations; for example, the number of samples and survey area were relatively small. However, our results imply the general tendency that TGC-susceptible *S.* Schwarzengrund has replaced TGC-resistant *Salmonella* as the predominant phenotype among broiler flocks in both Eastern and Western Japan. In this study, *bla*_CMY-2_ was detected as the resistance gene. Extended-spectrum β-lactamase-producing *Salmonella* strains carrying *bla*_TEM-52_ were reported for the first time from broiler chickens in Japan during 2004–2006^31^^)^. *Salmonella* strains harboring *bla*_CMY-2_, *bla*_TEM-20_, or *bla*_CTX-M-25_ appeared in 2007 and 2008 in Southern Japan^32^^)^. The prevalence of *bla*_TEM-52_ remarkably increased^32^^)^ and became predominant, followed by *bla*_CMY-2_, in 2010 and 2011^33^^)^. In retail chicken meat products, the first *Salmonella* spp. harboring *bla*_CMY-2_ were isolated in the mid 2000s^34^^)^. The prevalence of *bla*_CMY-2_ increased until 2011, and decreased after 2012, partially due to the change in the prevalence of TGC-resistant *Salmonella* (mainly *S.* Infantis) harboring *bla*_CMY-2_^10^^,^^20^^,^^35^^)^. Our results are consistent with these previous reports.

In conclusion, TGC-susceptible *Salmonella* has replaced the resistant phenotypes among broiler flocks in both Eastern and Western Japan, leading to a decreased prevalence of TGC-resistance in *Salmonella*. Chicken meat products could be cross-contaminated with *Salmonella* during the slaughtering process; however, reducing the prevalence of *Salmonella* in broiler flocks remains an important measure to decrease *Salmonella* infections in humans because the relationship between a reduction in the prevalence of *Salmonella*-contaminated chickens and the corresponding reduction in the risk of human illness was reported to be one-to-one,^12^^)^ and the prevalence of *Salmonella* in broiler flocks remains high (>70%) as revealed in this study. From a food safety perspective, continuous nationwide surveillance is needed to determine the transition of the prevalence, serovars, and antimicrobial resistance profiles of *Salmonella* in humans, chickens, and chicken meat products to identify, implement, and evaluate effective countermeasures for human salmonellosis.
